# House feeding pattern increased male yak fertility by improving gut microbiota and serum metabolites

**DOI:** 10.3389/fvets.2022.989908

**Published:** 2022-09-02

**Authors:** Yanbin Zhu, Xin Li, Lousang-zhaxi, Suolang-zhaxi, Suolang, Ciyang, Guangming Sun, Cidan-yangji, Basang-wangdui

**Affiliations:** ^1^Institute of Animal Science and Veterinary, Tibet Academy of Agricultural and Animal Husbandry Sciences, Lhasa, China; ^2^Linzhou Animal Husbandry and Veterinary Station, Lhasa, China

**Keywords:** yaks, house feeding, free range, metabolome, microbiota, semen quality, reproductive performance

## Abstract

Yaks usually live in an extremely harsh natural environment resulting in low reproductive performance, so the production of yak cannot meet local demand in China. In order to solve this problem, the experiment aims to explore the effect of different feeding modes on the semen quality of male yaks, so as to provide a theoretical basis for improving the yield of yaks in Tibet. We used the combined analysis of metabolomics and microbial sequencing to explore the underlying mechanisms that affect the differences in semen quality between the house feeding (HF) system and the free range (FR). The results showed that the sperm motility (*P* < 0.001) and sperm concentration (*P* < 0.05) in the HF group were significantly higher than the FR group, and the abnormal sperm rate (*P* < 0.01) in HF was significantly lower compared to FR. House feeding modes increased some beneficial materials in blood and testis especially some antioxidants, unsaturated fatty acids, and amino acids. House feeding group increased some gut microbiota at genus level namely *Rikenellaceae, Bacteroides, Prevotellaceae_UCG-004, Bacteroidales_RF16*, and *Alloprevotella, DgA-11*. It was interesting that blood metabolites, testicular metabolites, and fecal microbiota were well-correlated with sperm parameters. Meanwhile, the blood metabolites and testicular metabolites were well-correlated with microbes. The result indicated that the HF model was beneficial for yak semen quality by improving the gut microbiota and blood metabolism to increase yak fertility.

## Introduction

Yak is a unique livestock species on the Qinghai-Tibet Plateau, mainly distributed in Tibet, Qinghai, and other regions with an altitude of more than 3,000 m in China (1, 2). Due to its strong adaptability to harsh environments, yak has been becoming the most important dominant animal species in livestock husbandry on the Qinghai-Tibet Plateau (3, 4). Therefore, yak has an irreplaceable economic status, which has an important impact on the development of the yak industry and the promotion of economic development in China (5). However, the harsh natural environment in the plateau area greatly limits the reproductive performance of yak. The semen quality of the breeding male yak not only affects the feeding efficiency but also affects the production performance of the offspring (6). Therefore, improving the semen quality is a great significant strategy to improve the reproductive performance of yaks.

Currently, there are two feeding modes of yaks in Tibet. One is traditional full-grazing and the other is house feeding (HF). The yak raised by grazing mainly feeds on natural pasture, which is higher in crude fiber and lower in protein than other feeds (7). On the other hand, house-feeding yaks are mainly fed a complete mixed diet, which has a comprehensive nutritional level (8). As we know, different feeding patterns can affect animal performance due to differences in nutrient levels (9). At the same time, different feeding methods can also affect the meat quality of animals (10). Studies have shown that different feeding methods can affect the composition of gut microbiota and blood metabolites, and the proposed gut-testis axis reveals that microbes can affect male fertility (11, 12). In fact, a study has shown that gut microbes and blood metabolites can work together to regulate semen quality in animals (13). Therefore, yaks with different feeding patterns can improve reproductive performance by changing the diversity of gut microbiota and blood metabolome, which is crucial for yak production.

The current situation of yak farming in the Qinghai-Tibet Plateau mainly has the following problems to be overcome. First of all, yaks generally suffer from low fertility. Some data show that the average reproductive rate of yaks is only 48.61%, of which more than half are given birth every 2 or 3 years. In addition, more than 90% of postpartum yaks are not in heat during the estrus season in the same year (14, 15). Secondly, the fresh semen quality of male yaks is not higher enough compared to the boars, the artificial insemination technology is also not developed in the yak field (16). Therefore, this experiment combined metabolomics and microbial sequencing technology to explore the effects of different feeding modes on the reproductive performance of male yak, and to reveal the underlying mechanism, so as to provide a theoretical basis for improving the yield of yak in Tibet.

## Materials and methods

### Yaks and experimental design

All animal procedures were approved by the Animal Care and Use Committee of the Linzhou Animal Husbandry and Veterinary Station (LAHV2021-23). Twenty yaks (similar age and weight) were used in this investigation at Linzhou Animal Husbandry and Veterinary Station, Tibet, China. There were two groups: (1) free range (FR), 10 yaks fed for natural forage, the nutrition level is in Supplementary Table 5; (2) house feeding (HF), 10 yaks fed with a basal diet, the nutrition level is in Supplementary Table 6. Semen samples were collected by gloved-hand techniques. After collection, three semen parameters were assessed: sperm concentration, sperm motility, and abnormal sperm rate, according to the reported methods (17). Blood samples were harvested by venipuncture from the hindlimb vein of yaks. Each blood sample was then centrifuged at 3,000 × g for 10 min at 4°C to obtain a plasma sample and subsequently stored at −80°C until analysis. Feces were collected from the yak rectum and then stored at −80°C for subsequent microbiota analysis (18). Testicular samples were obtained during yak slaughter, and a portion was fixed with 4% paraformaldehyde for tissue sectioning. Another portion was stored in cryovials for testicular metabolome analysis, then stored in liquid nitrogen.

### Using computer-assisted sperm assay sperm motility analyzer to detect sperm parameters

Sperm motility, sperm concentration, and abnormal sperm rate were determined by the computer-assisted sperm assay (CASA) method according to World Health Organization guidelines (19). Yak semen was diluted and then incubated at 37.5°C for the 30 s, after that transferred sperm on a pre-warmed counting chamber (MICROPTIC S.L., Barcelona, Spain). Finally, we can obtain the phenotype data.

### Yak feces microbiota sequencing

The samples were analyzed by Shanghai OE Biotech. Co., Ltd. (Shanghai, China).

#### DNA extraction

Total genomic DNA of yak feces was isolated using an E.Z.N.A.® Stool DNA Kit (Omega Bio-tek Inc., USA) following the manufacturer's instructions. DNA quantity and quality were analyzed using NanoDrop 2000 (Thermo Scientific, USA) and 1% agarose gel.

#### Library preparation and sequencing

The V3–V4 region of the 16S rRNA gene was amplified using the primers 338F (5′-ACTCCTACGGGAGGCAGCAG-3′) and 806R (5′-GGACTACHVGGGTWTCTAAT-3′) with Barcode. The reaction system included 4 μl of 5 × FastPfu Buffer, 2 μl of 2.5 mM dNTPs, 0.8 μl of each primer (5 μM), 0.4 μl of FastPfu Polymerase, and 10 μl of DNA template. The reactions were performed based on GeneAmp® 9700 (Applied Biosystems, Foster City, CA, USA), and the processes briefly were as follows: the denaturation lasted for 3 min at 95°C followed by 27 cycles of 95°C for 30 s, 55°C for 30 s, and 72°C for 45 s, with a final extension of 10 min at 72°C. Meanwhile, the amplified fragments were determined by electrophoresis on a 2% agarose gel. Then, the products were purified with the AxyPrep DNA Gel Extraction Kit (Axygen Bioscience, CA, USA) according to the manufacturer's instructions.

#### Analysis of sequencing data

Operational taxonomic unit (OTU) abundance information was normalized using a standard sequence number corresponding to the sample with the least sequences. The alpha diversity indices were calculated with QIIME (Version 1.7). PLS-DA was performed using R software (Version 2.15.3).

### Plasma and testicular metabolites determined by LC&GC/MS

Yak plasma and testicular were collected and maintained at −80°C. The protein was removed from the samples before LC-MS/MS analysis with ACQUITY UPLC and AB Sciex Triple TOF 5600 (LC&GC/MS).

The conditions for HPLC were: ACQUITY UPLC BEH C18 column (100 × 2.1 mm, 1.7 μm), solvent A [aqueous solution with.1% (v/v) formic acid], and solvent B [acetonitrile with 0.1% (v/v) formic acid] with a gradient program: 0–2 min, 5–20% B; 2–4 min, 20–25% B; 4–9 min, 25–60% B; 9–17 min, 60–100% B; 17–19 min, 100% B; 19–19.1 min, 100–5% B; and 19.1–20.1 min, 5% B. The flow rate was set at 0.4 ml/min and 5 μl was injected. ESI was used in the mass spectrometry program. Progenesis QI version 2.3 (Nonlinear Dynamics, Newcastle, UK) was used to normalize the peaks. Human Metabolome Database (HMDB), Lipidmaps (version 2.3), and METLIN software were used to qualify the data.

### Testicular tissue sections preparation

Testicular tissue section prepared method followed by Luo et al. (20). Testicular tissue was immersed and fixed with Carnoy's solution for 24 h. Testicular samples were then removed from formalin and embedded in paraffin. Subsequently, the paraffin blocks were sectioned to obtain 5 μm thick sections using a semi-automatic microtome (LONGSHOU, China). Sections were stained with H&E and viewed under an optical microscope.

### Statistical analysis

The multivariate analyses of serum and testis metabolites were performed using the SIMCA 14.1 software package (V14.1, MKS Data Analytics Solutions, Umea, Sweden). The data were subjected to analyze the variance using SPSS (23.0). The student's *t*-test was used to analyze the differences between the two groups, and the results were presented as means ± SEM. The relationships among the metabolome, sperm parameters, and microbiota were explored using Pearson's correlation analysis. The results were drawn by using GraphPad 8. With ^*^ indicating a statistically significant difference (*p* < 0.05), ^**^ indicating a highly significant difference (*p* < 0.01), and ^***^ indicating a highly significant difference (*p* < 0.001).

## Results

### House feeding increased yaks' semen quality

**Figure 1:**
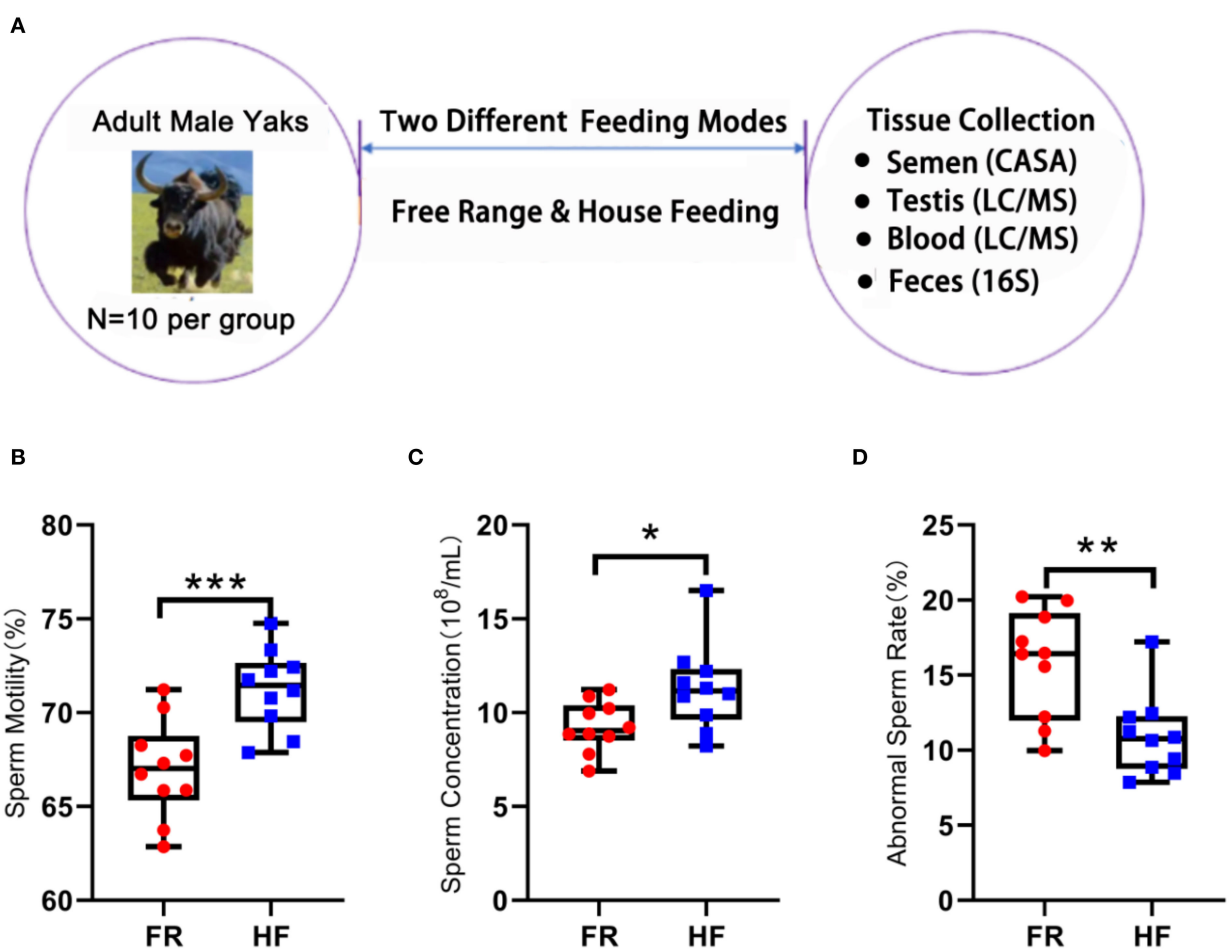
House feeding modes benefit yak semen quality. **(A)** Experimental design. **(B)** Sperm motility is determined by CASA, Y-axis = % of total sperm, X-axis = feeding modes. *n* = 10. ***P < 0.001. **(C)** Sperm concentration, Y-axis = sperm concentration (10^8^/ml), X-axis = feeding modes *n* = 10. *P < 0.05. **(D)** Abnormal sperm rate. Y-axis = % of abnormal sperm, X-axis = feeding modes. **P < 0.01.

Twenty adult yaks with similar age and weight in different feeding patterns were used in the current investigation: 10 yaks for HF and 10 for FR. We collected semen, testis, blood, and feces as samples to analyze (Figure 1A), and the HF group showed significantly increased sperm motility (Figure 1B; *p* < 0.001). The sperm density showed a significantly increased in HF over FR (Figure 1C; *p* < 0.05). However, the abnormal sperm rate in the FR was higher than HF (Figure 1D; *p* < 0.01). Therefore, the data suggested that HF mode is better than FR because the HF can improve yak semen quality and fertility.

### House feeding benefited testicular histomorphological integrity

**Figure 2:**
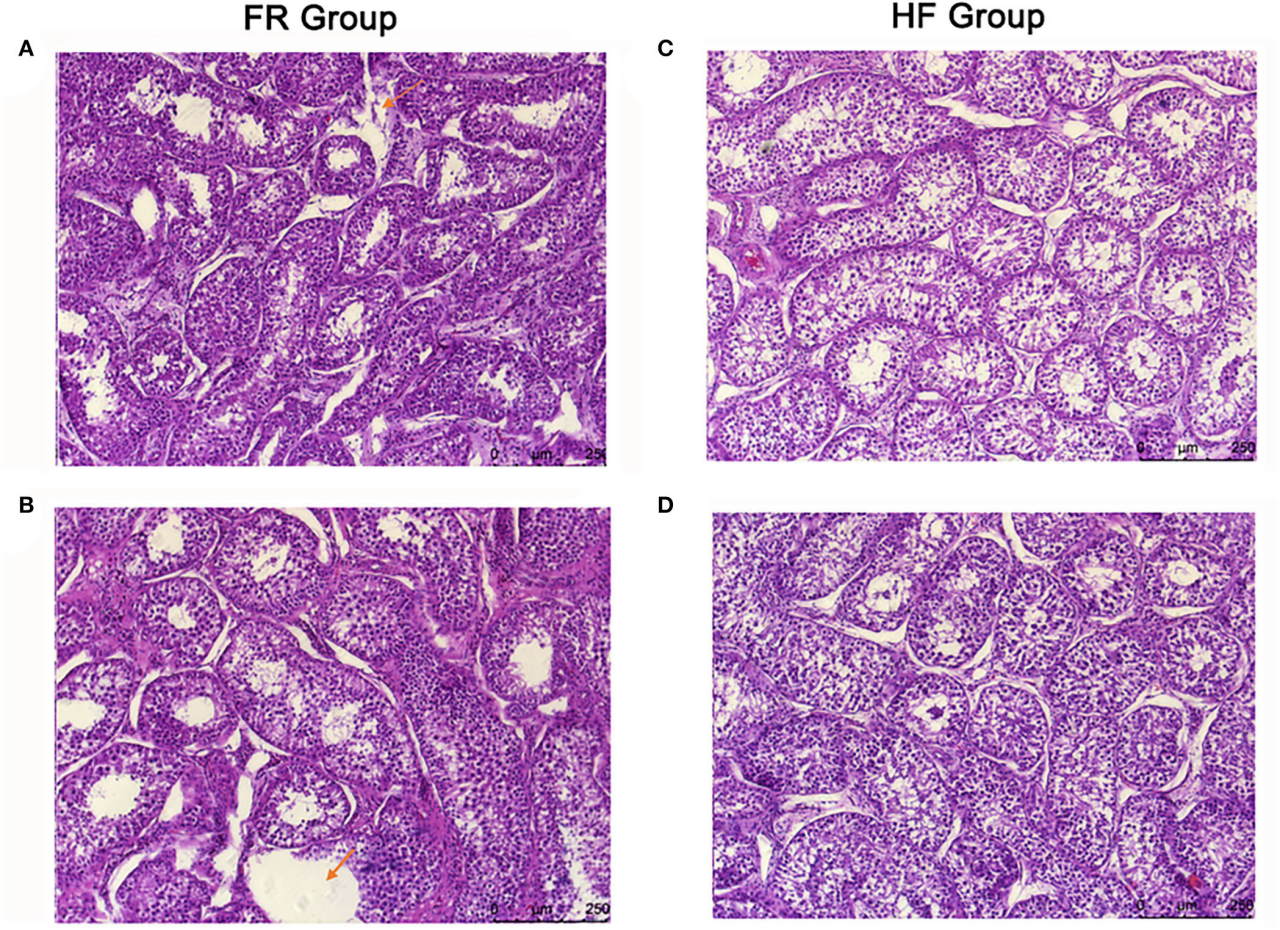
**(A,B)** H&E-stained sections of yak testis in free range group under 10x microscope. The arrow in **(A)** means part of the connective tissue was broken in the FR group. The arrow in **(B)** means the space between the seminiferous tubules was enlarged, and the seminiferous tubules were also loosely arranged and structurally disordered. **(C,D)** H&E-stained sections of yak testis in house feeding group under 10x microscope.

The testicular tissue sections of yak were made by H&E staining. It was found that the space between the seminiferous tubules was enlarged, the seminiferous tubules were also loosely arranged and structurally disordered, and part of the connective tissue was broken in the FR group (Figures 2A,B). On the contrary, the testicular tissue of the yak in the HF group was closely arranged, and the seminiferous tubule structure was intact without lesions (Figures 2C,D).

### House feeding modes improved yaks blood metabolites

**Figure 3:**
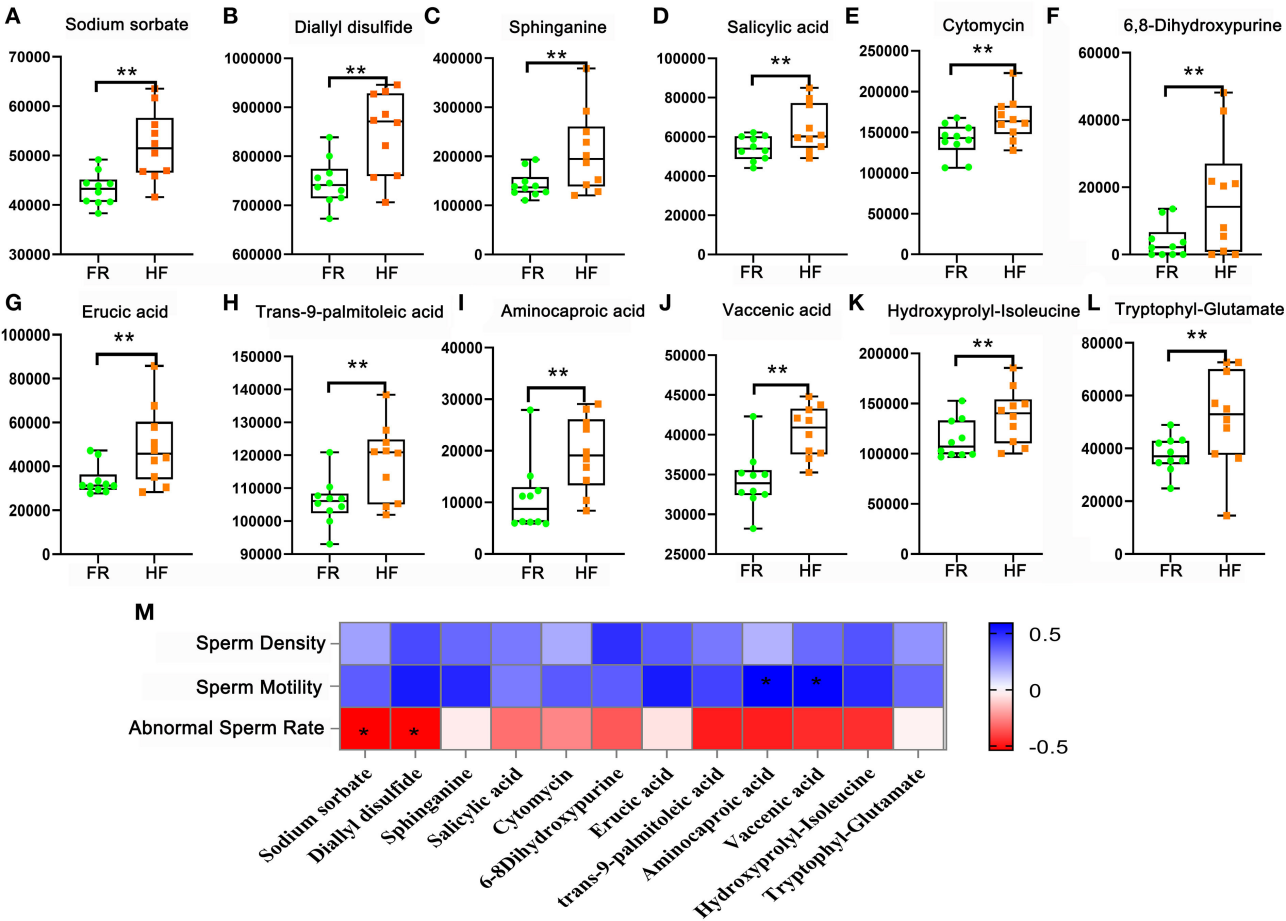
House feeding modes benefited yak blood metabolome. **(A–L)** Increased blood metabolites Y-axis = relative amount, X-axis = feeding modes **P < 0.01. **(M)** Correlation of blood metabolites and sperm concentration, motility, and abnormal sperm rate. *P < 0.05.

In this part, we used LC&GC/MS to detect blood plasma, there were 165 metabolites detected in blood plasma samples (Supplementary Table 1). Here, we found out that 12 metabolites were significantly different in HF compared with FR. Some antioxidants in the HF group were significantly higher than the FR group such as Sodium sorbate, Diallyl disulfide, Sphinganine, Salicylic acid, Cytomycin, and 6,8-Dihydroxypurine (Figures 3A–F). At the same time, some unsaturated fatty acids in the HF group were significantly higher than in the FR group, they were Erucic acid, trans-9-palmitoleic acid, Aminocaproic acid, and Vaccenic acid (Figures 3G–J). Functional enrichment of these metabolites showed that they were involved in amino acid metabolites for example Hydroxyprolyl-Isoleucine and Tryptophyl-Glutamate in HF were significantly higher than in FR (Figures 3K,L). As we all know that antioxidants, unsaturated fatty acids, and amino acids are all beneficial materials to improve blood health. Meanwhile, these metabolites were well-correlated with sperm parameters (Figure 3M). We can see that fatty acids (Aminocaproic acid and Vaccenic acid) were positively correlated with sperm motility. However, Sodium sorbate and Diallyl disulfide were negatively correlated with abnormal sperm rates. The data suggested that blood metabolites may contribute to semen quality changeable.

### House feeding pattern improved yak testicular metabolome

**Figure 4:**
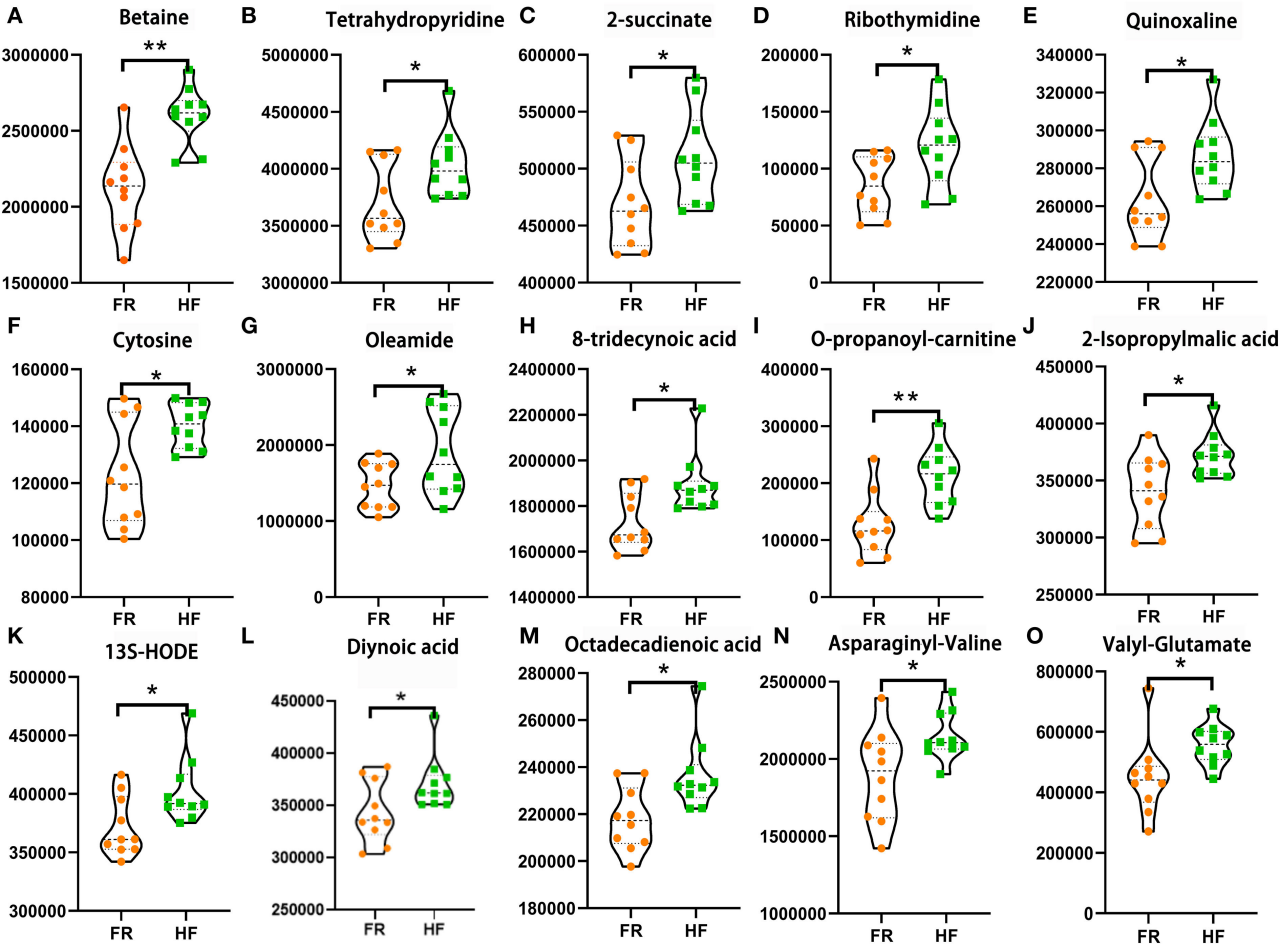
House feeding modes benefited yak testicular metabolome. **(A–O)** Increased testicular metabolites Y-axis = relative amount, X-axis = feeding modes **P < 0.01; *P < 0.05.

**Figure 5:**
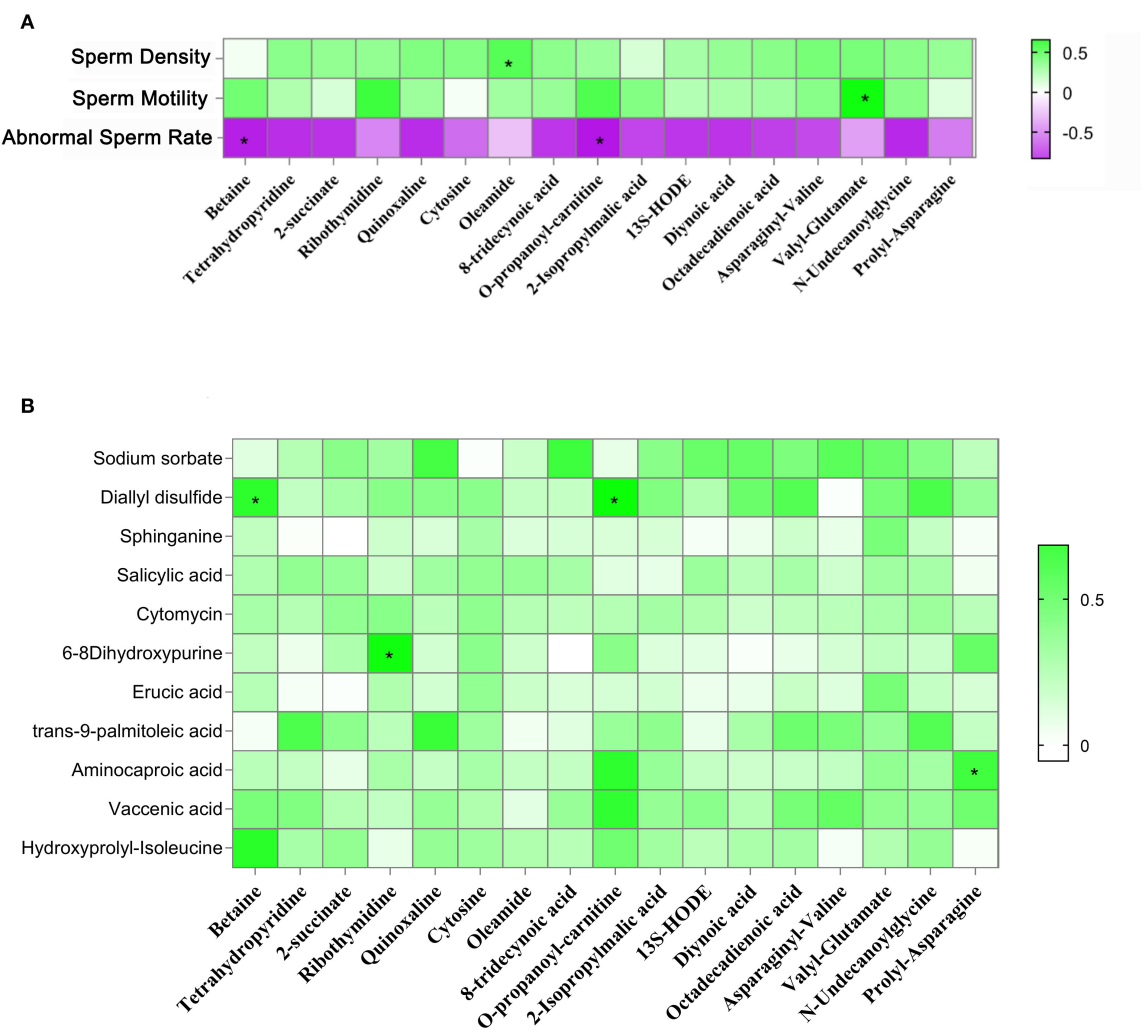
**(A)** Correlation between testicular metabolites and sperm parameters. **(B)** Correlation between blood metabolites and testicular metabolites. *P < 0.05.

In this section, we used LC&GC/MS to investigate yak testicular metabolites. There were 117 metabolites detected in the yak testicular samples (Supplementary Table 2). According to the raw data, 15 metabolites were significantly higher in the HF group compared to the FR group. Six of the significantly increased metabolites are shown in Figures 4A–F which are reported to be beneficial to animals because of their antioxidant activity. Some of the significantly increased metabolites are shown in Figures 4G–M. They are all fatty acids. As known, unsaturated fatty acids can be used during the spermatogenesis procedure. Some amino acids in the HF group were also significantly higher than in the FR group (Figures 4N,O). These metabolites were well-correlated with sperm motility, sperm density, and abnormal sperm rate (Figure 5A), from the figure we can see that all testicular metabolites were negatively correlated with abnormal sperm rate, especially betaine and O-propanoyl-carnitine. At the same time, the testicular metabolites were well-correlated with sperm density and motility, the Oleamide was positively correlated with sperm density and Valyl-Glutamate was also positively correlated with sperm motility. As shown in Figure 5B the testicular metabolites had well-correlated with blood metabolites. The data suggested that blood may contribute to changing testicular metabolites to improve semen quality in HF modes.

### House feeding patterns benefited gut microbiota

**Figure 6:**
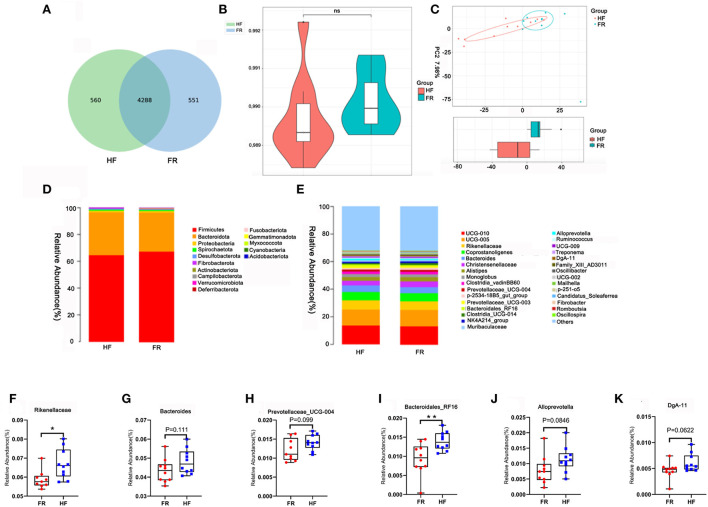
House feeding modes improved yak gut microbiota. **(A)** The Wayne figures of fecal microbiota. **(B)** The Chao 1 index of fecal microbiota. **(C)** PC analysis of fecal microbiota at the OUT level. **(D)** Difference bacterial abundance at the phylum level. **(E)** The relative abundances of fecal microbiota at the genus level. **(F–K)** The relative abundances of six different microbes in the genus level, which were increased in house feeding modes. **P < 0.01; *P < 0.05.

**Figure 7:**
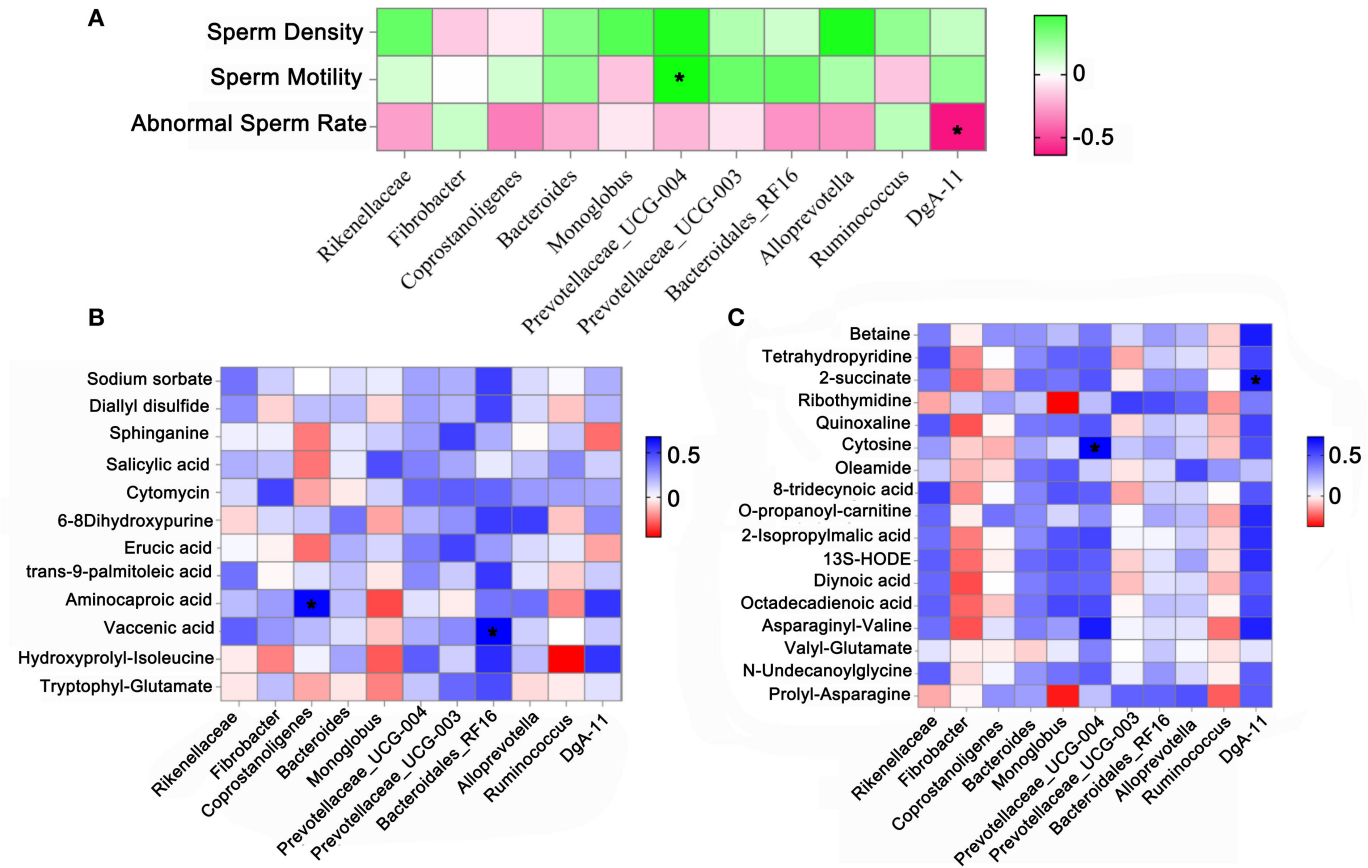
**(A)** Correlation between gut microbes and sperm parameters. **(B)** Correlation between blood metabolites and gut microbes. **(C)** Correlation between testicular metabolites and gut microbes. *P < 0.05.

Fecal samples were determined to identify the differences between the HF group and the FR group on gut microbiota, which may contribute to altering blood metabolites and testicular metabolites. The data showed that feeding modes affected some of the microbiota in yak fecal samples (Supplementary Tables 3, 4). There were 4,848 microbes in the HF group and 4,839 in the FR group. There were 560 unique microbes in the HF group, while 551 were found in the FR group (Figure 6A). However, the α-diversity indicated by Chao1 was not significant (Figure 6B). Meanwhile, the β-diversity was shown in Figure 6C. The microbiota in fecal samples represents the microbiota in the large intestine. At the phylum level, compared to the FR group, the HF group had increased levels of *Bacteroidetes*, decreased *Firmicutes* (Figure 6D), and an increased ratio of *Bacteroidetes/Firmicutes*. Moreover, at the Genus level, we enrich the top 30 microbes (Figure 6E), among them we found out 6 different microbes in the HF group were higher than in the FR group, some of them are significant and some are not, but had the tendency (Figures 6F–K). The microbiota and sperm parameters were well-correlated. The *Prevotellaceae_UCG-004* was positively correlated with sperm motility; Meanwhile, *DgA-11* was negatively correlated with abnormal sperm rate (Figure 7A).

Furthermore, blood metabolites and fecal microbiota were well-correlated (Figure 7B). Blood metabolites, Aminocaproic acid, increased in the HF group and were positively correlated with the beneficial microbiotas *Coprostanoligenes*. Similarly, Vaccenic acid was positively correlated with beneficial microbiotas *Bacteroidales_RF16*. The testicular metabolites called cytosine which was increased in the HF group was positively correlated with the beneficial microbiotas *Prevotellaceae_UCG-004*, the 2-succinate was also positively correlated with *DgA-11* (Figure 7C).

## Discussion

There is no doubt that gut microbiota has been becoming a hot topic in recent years because of many physiological roles; the presence of the gut-testis axis was demonstrated, suggesting that gut microbiota can influence male fertility. Previously, people found that some natural plant extracts benefits gut microbiota by increasing “beneficial” bacteria while decreasing “harmful” bacteria in murine small intestines to rescue semen quality of mice (21). In our experiment, we found six different kinds of microbes increased in the HF group in the genus level, *Rikenellaceae* was significantly higher than the FR group. The study has shown that it was a kind of beneficial bacteria in Bull fertility (22), and it can influence the production of Butyric acid that was used for spermatogenesis. Another increased microbes were *Bacteroidales_RF16*, it was also a beneficial bacteria because it related to reducing heat stress in a pig model (23), adding forage can also increase the microbes in yaks due to its high rate of digesting cellulose (24). However, no research has shown that the bacteria is related to male reproductive performance. In this experiment, we speculated based on the phenotype that it may be related to the reproductive performance of yaks, which needs further experimental verification.

Gut microbial metabolic nutrients in the intestine can regulate intestinal metabolites to influence the blood metabolome (25, 26). In turn, while blood is cycled through body organs, blood metabolites can influence their development as feedback (27). As we know that metabolic regulation is essential for spermatogenesis (28–30), cholesterol and lipid homeostasis play a vital role in male fecundity (31–35). In our investigation, we analyzed blood metabolites, it was found that the content of antioxidants was increased in the blood. These antioxidants can improve the blood of yaks very well so that they can adapt to the high-altitude environment, which is beneficial to the health of yaks. Sodium sorbate was significantly higher in the HF group, the study had shown that it can be used as a safe food additive in diet to ferment corn silage (36, 37). In addition, unsaturated fatty acids and amino acid derivatives were increased in blood metabolites of yaks. The utilization of fatty acids and amino acids is essential during spermatogenesis (38–42), so the blood levels of these two nutrients were significantly increased in the house-feeding group, that is consistent with the phenotypic data. Blood metabolites are well-correlated with gut microbiota, so the semen quality of yaks may be regulated by the combination of blood metabolites and gut microbiota.

Testicular metabolome and tissue sections can reflect the physiological condition of the testis (43, 44). It is well-known that the role of the testis is mainly reflected in two aspects. On the one hand, the testis has the function of spermatogenesis (45). Sperm is produced in the epithelial cells in the testis, then it is transported to the seminal vesicles through the ureters for temporary storage (46). On the other hand, the testes produce androgens, which are important for maintaining male sexual characteristics (47–49). In our research, we found out that the testicular metabolites betaine significantly increased in the HF group, betaine has a regulatory effect on boar semen quality and it can be used as an osmoprotectant to increase sperm concentration (50). When the addition rate of betaine was 0.63 and 1.26%, the sperm density increased by 6 and 13%, respectively. Therefore, the addition of betaine to the diet at 0.63% increased sperm density without negatively affecting semen quality (51). Here, we may suppose HF mode can increase the level of betaine in the testis to increase the yak semen quality. Meanwhile, we also found out some fatty acids and amino acid levels increased in the HF group, they were Oleamide, 8-tridecynoic acid, and Octadecadienoic acid (52). There are double bonds in their structure, which have good biological activity and could be used by yaks. Apart from that, they are all beneficial to spermatogenesis (53, 54). According to the testicular tissue section, we can find that the seminiferous tubules in the free-range group have necrosis, the structure is disordered, part of the connective tissue is broken, and the space between the seminiferous tubules is widened and the arrangement is loose. The increased antioxidants are benefitted to reduce the reactive oxygen species in the epididymis (55). The increased fatty acids and amino acids are all beneficial to sperm formation (56). Therefore, combining the testicular metabolome and tissue sections, we can conclude that HF is more conducive to protecting the yaks' testis and improving its semen quality. There is also a good correlation among the testicular metabolome, microbiome, and sperm parameters, which may also indicate the existence of the gut-testis axis.

Currently, in the Tibet region of China, the yak is the main economic animal (57). Ninety percent of the world's yak meat is produced in the Qinghai-Tibet Plateau of China (58). However, the growth of yak is relatively slow and the reproductive cycle is longer than that of cattle, so the yield of yak meat is still not high enough (59). Especially the reproductive performance, the sperm motility of yak is lower than cattle (60), which may be due to long-term living in high-altitude areas. Most yaks are raised on a free-range model. The free-range mode may lead to the insufficient nutritional intake of yaks, and there is insufficient nutrient to use during spermatogenesis, resulting in poor semen quality. Conversely, yaks raised in house-feeding mode are fed a basal diet with balanced nutrition, and their sperm motility can exceed 70% (61), which is consistent with our study. Since the artificial insemination technology of yaks is not mature enough compared to pigs, changing the feeding mode is an effective way to improve the semen quality of yaks. In this way, it can not only improve the pregnancy rate of female yaks but also increase the number of fetuses.

## Conclusion

In summary, we found that the HF model was beneficial to the semen quality of yaks by improving the gut microbiota and blood metabolism. This kind of feeding mode can be promoted so that the production of yak meat in Tibet can be further increased for global consumption.

## Data availability statement

The data presented in the study are deposited in the NCBI SRA database with accession number PRJNA871215.

## Ethics statement

The animal study was reviewed and approved by the Animal Care and Use Committee of the Linzhou Animal Husbandry and Veterinary Station (LAHV2021-23).

## Author contributions

YZ and XL performed the experiments and analyzed the data. L-z, S-z, S, and C designed and supervised the study. YZ, GS, and C-y wrote the manuscript. B-w revised the manuscript. All the authors edited the manuscript and approved the final manuscript.

## Funding

This research was supported by the Breeding and Efficient Propagation of Yaks in Gesangtang of Linzhou County (QYXTZX-LS2020-01), Seed Industry Innovation and Healthy Breeding of Yaks (XZ202101ZD0002N), and National Meat Yaks Industry Technology System (CARS-37).

## Conflict of interest

The authors declare that the research was conducted in the absence of any commercial or financial relationships that could be construed as a potential conflict of interest.

## Publisher's note

All claims expressed in this article are solely those of the authors and do not necessarily represent those of their affiliated organizations, or those of the publisher, the editors and the reviewers. Any product that may be evaluated in this article, or claim that may be made by its manufacturer, is not guaranteed or endorsed by the publisher.

## Supplementary material

Supplementary Table 1 Raw data of blood metablome (total).

Supplementary Table 2 Raw data of testicular metablome (total).

Supplementary Table 3 16S raw data on phylum level.

Supplementary Table 4 16S raw data on genus level.

Supplementary Table 5 Nutrition level of nature pasture.

Supplementary Table 6 Nutrition level of basal diet.
